# MicroRNA-145-5p regulates the proliferation of epithelial ovarian cancer cells via targeting SMAD4

**DOI:** 10.1186/s13048-020-00656-1

**Published:** 2020-05-04

**Authors:** Jie Zhou, Xiyi Zhang, Weiling Li, Yuanyuan Chen

**Affiliations:** Department of Obstetrics and Gynecology, Xi’an Gaoxin Hospital, No. 16 Tuanjie South Road, Xi’an, 710075 Shaanxi China

**Keywords:** MiR-145-5p, Epithelial ovarian cancer, SMAD4, Migration, Apoptosis, Proliferation

## Abstract

**Background:**

Epithelial ovarian cancer (EOC) is one of the most prevalent malignancies affecting females worldwide; however, its etiology mechanism remains unclear. In various malignancies, miR-145-5p is a widely accepted and versatile miRNA. Therefore, our research focused on exploring the activity and etiology of miR-145-5p in the modulation of metastasis, migration, and proliferation of EOC cells. The direct reactions between the 3′UTRs of SMAD4 mRNA and miR-145-5p were verified using dual luciferase reporter test. SKOV-3 cells were subsequently transfected using miR-145-5p mimics. Cell migration, death, and proliferation were evaluated using MTT, flow cytometry, and Transwell test. In addition, SMAD4 transcription and translation were evaluated using qRT-PCR and Western blot.

**Results:**

We found that miR-145-5p expression was repressed prevalently in EOC tissues, apart from SMAD4 upregulation. Excessive miR-145-5p expression remarkably reinforced EOC cell death and repressed EOC cell proliferation. Furthermore, upregulated miR-145-5p expression noticeably repressed migration via MMP-2 and MMP-9 downregulation. Moreover, SMAD4 was downregulated via miR-145-5p transfection. The dual luciferase test revealed that miR-145-5p directly targeted SMAD4.

**Conclusions:**

Our research suggests that miR-145-5p serves as a malignancy repressor and exerts an essential impact on inhibiting malignancy generation and reinforcing EOC death via targeting SMAD4. MiR-145-5p application could serve as a promising strategy to treat EOC.

## Introduction

Epithelial ovarian cancer (EOC) is the dominant contributor to gynecologic malignancy-related death in females with poor prognosis, with an annual mortality of approximately 125,000 [1]. Unfortunately, only 19% of the total ovarian malignancy cases are identified early. In most women, it is diagnosed at an advanced stage, which largely explains the poor prognosis of this malignancy. Germline mutations of the genes BRCA1 and BRCA2, which encode proteins essential for the repair of double-strand DNA breaks through homologous recombination, lead to increased cancer predisposition. BRCA mutations are present in approximately 14% of epithelial ovarian cancers. The poor mortality of this illness is attributable to diagnosis made at the terminal stage, accounting for approximately 70% of the total ovarian malignancies [2, 3]. Contemporary research suggests that every histologic type of EOC is related to different morphologic and molecular mutations, such as endometroid, clear-cell, mucinous, and serous carcinomas [4–6]. Consequently, further research on the etiology and mechanism that reinforce ovarian malignancies are required to explore reliable predictors as well as innovative drugs to develop an efficient personalized treatment.

MicroRNAs (miRNAs) are a group of small single-stranded RNAs consisting of 22 nucleotides with a typical hairpin secondary structure [7]. They modulate gene silencing via directly targeting at an mRNA for degeneration or repressing translation [8]. A change in miRNA expression is related to different kinds of malignancies, such as EOC [9–11]. MiRNAs serve as the essential modulators of multiple fundamental biological reactions, such as those associated with malignancy generation [12]. Reportedly, miRNAs exert an essential impact on cell proliferation, apoptosis, and differentiation [13–15]. Primary malignancies, as well as cell lines, display a considerable expression of multiple malfunctioning miRNAs in comparison with normal tissues [16]. For example, miR-34 located at the downstream of p53 could modulate proliferation repression, cell death, and senescence stimulation in multiple cell types [17, 18]. Numerous miRNAs display malignancy-repressing capabilities where an abnormal expression of miRNAs in malignancies could offer a promising treatment strategy [19]. Particularly, excessive miR-145-5p expression can repress serous EOC progression [20]. Nevertheless, we need to further elucidate the functions and mechanism of miR-145-5p.

In the present study, we explored the expression and activity of miR-145-5p in EOC. We revealed that EOC was possibly modulated via miR-145-5p. When compared with that in normal ovarian tissues (NOT), miR-145-5p was constantly repressed in EOC tissues. However, excessive miR-145-5p expression repressed EOC cell proliferation and migration as well as triggered EOC cell death. Such activities are related to the inhibition of SMAD4 expression.

## Materials and methods

### EOC cell lines and tissues

We acquired 18 samples of EOC tissues and another 18 samples of NOT (surrounding the malignancies) from the Xi^’^an Gaoxin Hospital. The diagnosis of every participant was histopathologically verified. None of the patients underwent previous malignancy-counteracting treatment or displayed distant metastasis. Every specimen was obtained between 2015 and 2017, and it was fixed using formalin with the approval from the local ethics committee. The study was approved by the Ethics Committee of the Xi^’^an Gaoxin Hospital. Fully informed written consent was acquired from every patient.

EOC cell lines (SKOV-3) were purchased from the Chinese Academy of Science and were cultured in RPMI-1640 (Gibco, Carlsbad, CA, USA) medium supplemented with 10% or 20% fetal bovine serum (FBS, Gibco) and 1% penicillin (Sigma-Aldrich, Inc., St-Louis, MO, USA) in a humidified 5% CO2 incubator at 37 °C.

### Cell transfection

SKOV-3 cells were transfected using mature miR-145-5p mimics to explore the modulating impact of miR-145-5p. Lipofectamine 3000 reagent was applied to the transfection, as per the manufacturer’s instructions. Nonhomologous miRNA mimics served as the negative control (NC). Cells underwent trypsinization, and 24 h after transfection, they were harvested for cell death and proliferation test. MiR-145-5p mimics and NC were purchased from RiboBio Co., Ltd. (Guangzhou, China).

### RNA extraction and real-time PCR

We isolated total RNA via TRIzol and purified using the RNeasy Mini Kit (Qiagen, Hilden, Germany). We used Superscript III Kit (Life Technologies) to perform reverse transcription. Thereafter, complementary DNAs were assessed using qRT-PCR. Related transcriptions were quantified and assessed with qRT-PCR using the SYBR Green PCR Supermix Kit. Procedures were performed at least thrice for every specimen.

### Cell viability assay

Cell survival was assessed using MTT test. Plates containing 96 wells were used to plant the cells (5 × 10^4^ cells/mL) that were cultivated under the conditions of 5% CO_2_ and 37 °C. MTT test was conducted at 48 h after transfection. Cell survival was assessed by supplementing 10 μL of MTT to every well. Cells were incubated at 37 °C for 4 h, following which they were evaluated using a microplate reader at 570 nm (Thermo Scientific). Procedures were performed thrice independently.

### Assessment of cell death

Apoptosis was evaluated using flow cytometry (FC). After the cells were acquired, they were washed twice using cold PBS. The supernatant was discarded after 5 min of centrifugation at 100 rpm. The pellet was re-suspended in a binding buffer. FITC-Annexin V and propidium iodide (PI) were used for supplementation. The mixture was incubated at room temperature for 10 min. Fluorescence signals were evaluated using FACScan flow cytometer.

### TUNEL test

Using 4% paraformaldehyde, SKOV-3 cells were fixed on the slide. Dead cells were labeled using TUNEL.

### Cell migration test

Cell migration was evaluated via the Transwell test. Briefly, 50,000 cells in DMEM containing 1 μg/mL of mitomycin C without serum were planted on the top well of 24-well poly-carbonate Transwell filters (Millipore, Bedford, MA, USA), whereas the bottom well was supplemented with DMEM with 10% fetal bovine serum. After 48 h of incubation, we removed the cells in the top well, whereas those on the bottom surface were fixed, stained, and quantified.

### Dual luciferase reporter test

Our research examined whether excessive miR-145-5p expression repressed the firefly luciferase function in SKOV-3 cells. Such cells were previously transducted using luciferase plasmids with SMAD4 3′UTR to examine whether miR-145-5p could directly modulate SMAD4 expression. Briefly, pGL3-SMAD4 was cotransfected using miR-145-5p mimics or a repressor into cultivated SKOV-3 cells using the Neon Transfection Kit (Lonza). Luciferase function was assessed using the Dual-Luciferase Reporter Assay System (Promega).

### Western blot

Proteins were quantified by Bradford test (Bio-Rad, Hercules, CA, USA) and standard SDS–PAGE. These proteins were then isolated on 8–15% Tris–HCl polyacrylamide gels (Bio-Rad), following which they were transferred to polyvinylidene difluoride. Thereafter, the blots were incubated overnight using particular primary antibodies (anti-p21, anti-cyclinD, anti-MMP-9, anti-SMAD4, anti-p27, anti-MMP-2, and anti-β-actin from Cell Signaling Technology, USA) within TBST at 4 °C. Re-incubation was performed using secondary antibodies in conjugation with horseradish peroxidase. Enhanced chemiluminescence plus a detection reagent (Pierce, Rockford, USA) was employed to evaluate immunoreactive signals.

### Statistical analysis

The outcome is presented as mean ± SEM. Difference between various groups was evaluated via unequal-variance Student’s *t*-test (two-tailed) or ANOVA, followed by Tukey’s post hoc test. *P* < 0.05 is considered significant.

## Results

### MiR-145-5p was downregulated in EOC tissues

**Fig. 1:**
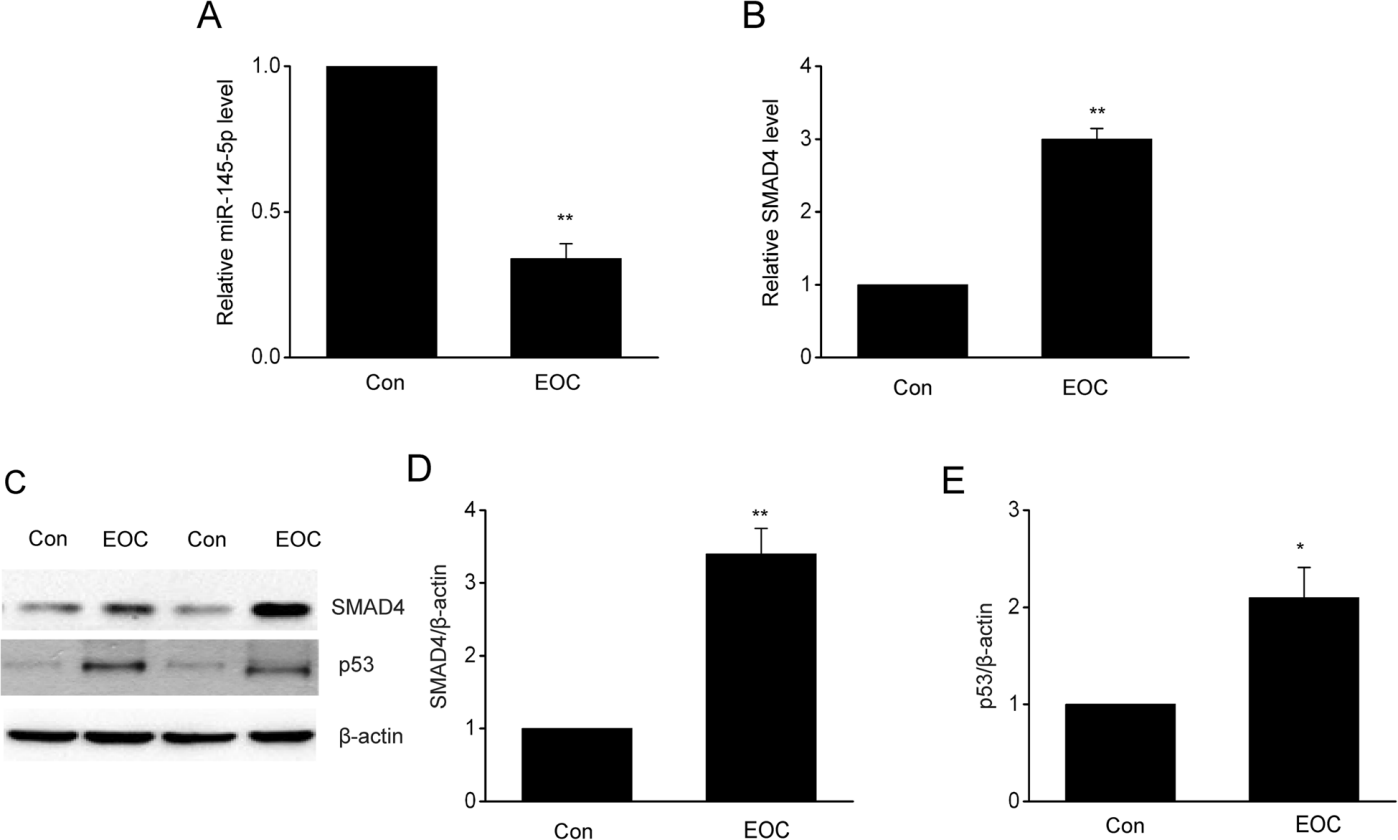
MiR-145-5p expression was inhibited in epithelial ovarian cancer (EOC). **a-b**, Expression of miR-145-5p (**a**) and SMAD4 (**b**) in EOC and healthy ovarian tissues (surrounding the malignancies; Con) was assessed using qRT-PCR. **c-e**, Western blots (**c**) and the quantitative evaluation of SMAD4 (**d**) and p53 (**e**) in EOC and tumor adjacent tissues. Data are presented as means ± SEM, *n* = 18. ^**^P < 0.01, compared with the NC group

The qRT-PCR that was performed to explore miR-145-5p in EOC tissues revealed that the transcription of miR-145-5p was repressed in EOC tissues compared with nonmalignant ones (Fig. 1). Moreover, SMAD4 transcription and translation were noticeably upregulated in EOC tissues compared with nonmalignant ones. In addition, the expression of p53 was also increased in EOC tissues. Overall, miR-145-5p was often repressed in EOC and remarkably modulated SMAD4.

### MiR-145-5p targeted SMAD4 in SKOV-3 cells

**Fig. 2:**
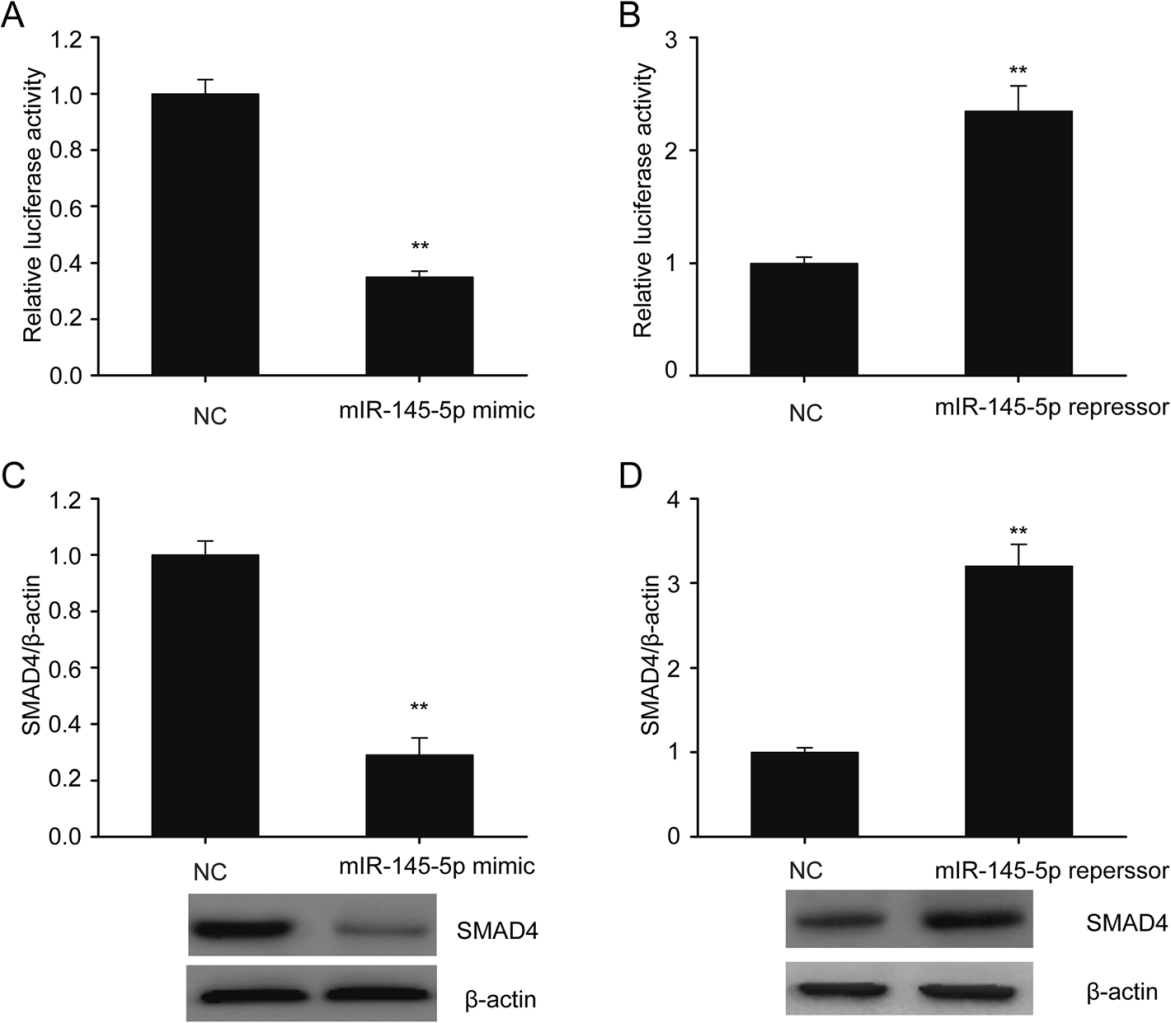
MiR-145-5p targeted SMAD4. **a-b**, Associations between the 3′UTRs of SMAD4 transcripts and miR-145-5p were assessed using dual luciferase test. **c-d**, Immunoblots and the quantitative evaluation of SMAD4 in SKOV-3 cells that were transfected for 24 h using miR-145-5p mimic (**c**) or miR-145-5p repressor (**d**). Data are presented as means ± SEM, *n* = 3. ^**^P < 0.01, compared with the NC group

Bioinformatics analysis indicated that SMAD4 could be targeted via miR-145-5p. The SMAD4 3′UTR function of SKOV-3 cells was inhibited via excessive miR-145-5p expression but was enhanced via miR-145-5p downregulation (Fig. 2a-b). Therefore, miR-145-5p probably regulated SMAD4 directly via 3′UTR binding. Moreover, excessive miR-145-5p expression repressed SMAD4 translation. However, miR-145-5p repression reinforced SMAD4 translation of SKOV-3 cells (Fig. 2c-d).

### MiR-145-5p repressed SKOV-3 cell proliferation

**Fig. 3:**
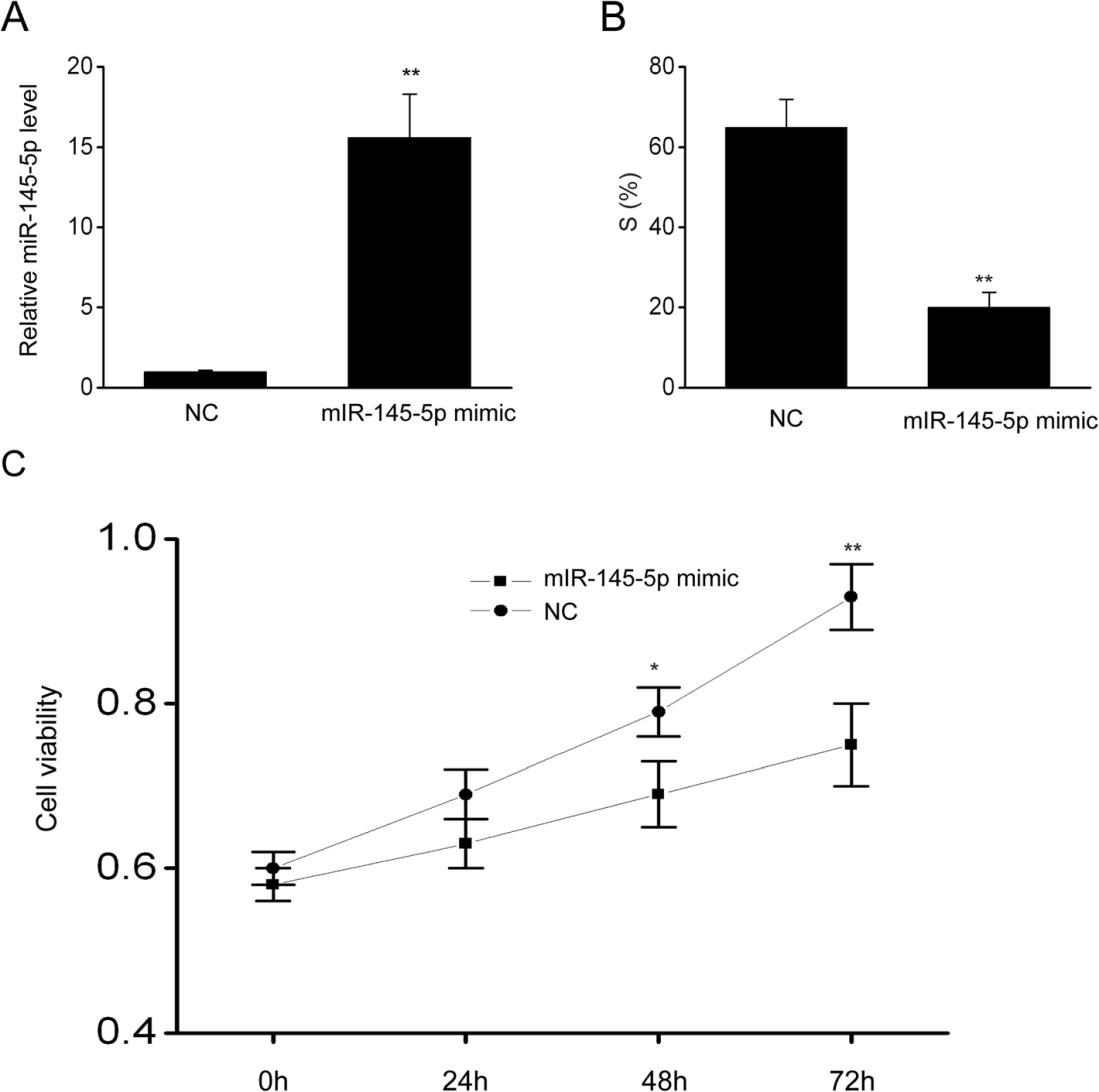
MiR-145-5p repressed the proliferation of EOC cells. **a**, MiR-145-5p expression in SKOV-3 cells following transient transfection using miR-145-5p mimic or NC. **b**, miR-145-5p prohibited SKOV-3 cell proliferation, as shown using FC. **c**, Excessive miR-145-5p expression repressed such proliferation, as demonstrated using MTT test. Data are presented as means ± SEM, n = 3. ^**^P < 0.01, ^*^P < 0.05, compared with the NC group

SKOV-3 cells were transfected using miR-145-5p mimics, and their proliferation was assessed using MTT and FC to explore the contribution of miR-145-5p to EOC cell proliferation. MiR-145-5p concentration was remarkably elevated in SKOV-3 cells in comparison with that in NC (Fig. 3a). Further, FC revealed that the S phase of SKOV-3 cells was repressed in specimens supplemented with miR-145-5p mimic compared with that of NC (Fig. 3b). Moreover, MTT test proved that SKOV-3 cell proliferation was remarkably repressed following an excessive miR-145-5p expression in comparison with NC cell proliferation (Fig. 3c), suggesting that miR-145-5p repressed EOC cell proliferation.

### MiR-145-5p repressed the expression of cell cycle-related proteins in SKOV-3 cells

**Fig. 4:**
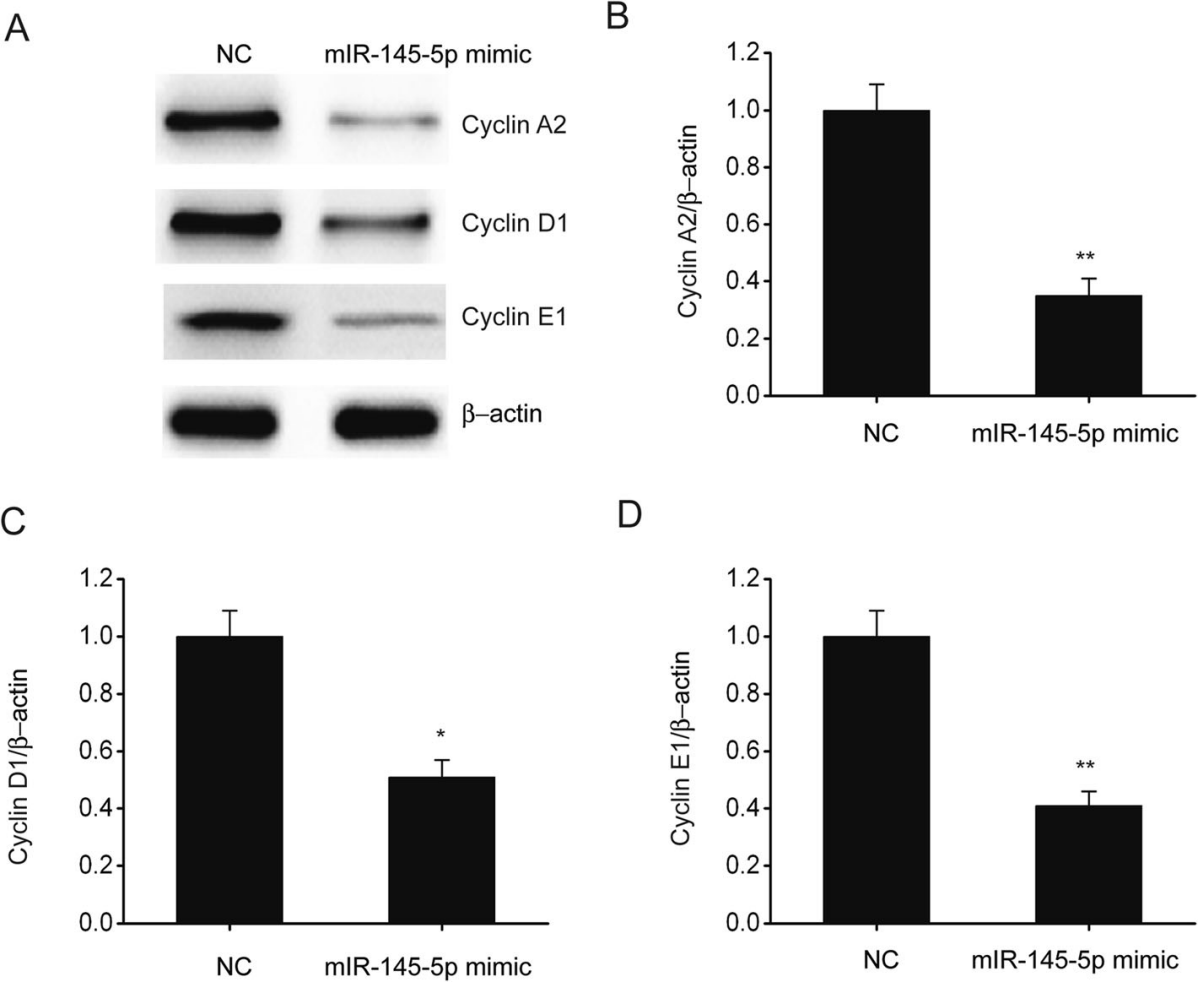
MiR-145-5p prohibited the expression of cell cycle-related proteins in SKOV-3 cells. **a-d**, Immunoblots (**a**) and the quantitative evaluation of cyclins A2 (**b**), D1 (**c**), and E1 (**d**) in SKOV-3 cells that were transfected for 24 h using miR-145-5p mimic or NC. Data are presented as means ± SEM, n = 3. ^**^P < 0.01, compared with the NC group

Previous research has demonstrated that cyclins E1, A2, and D1 contributes to the proliferation modulation of EOC [21]. In the current research, we investigated whether miR-145-5p repressed EOC cell proliferation using the abovementioned proteins. We observed that miR-145-5p downregulated the translation of these cell cycle-modulating proteins in SKOV-3 cells (Fig. 4). Therefore, miR-145-5p repressed EOC cell proliferation by repressing the cyclins E1, A2, and D1.

### MiR-145-5p reinforced EOC cell death

**Fig. 5:**
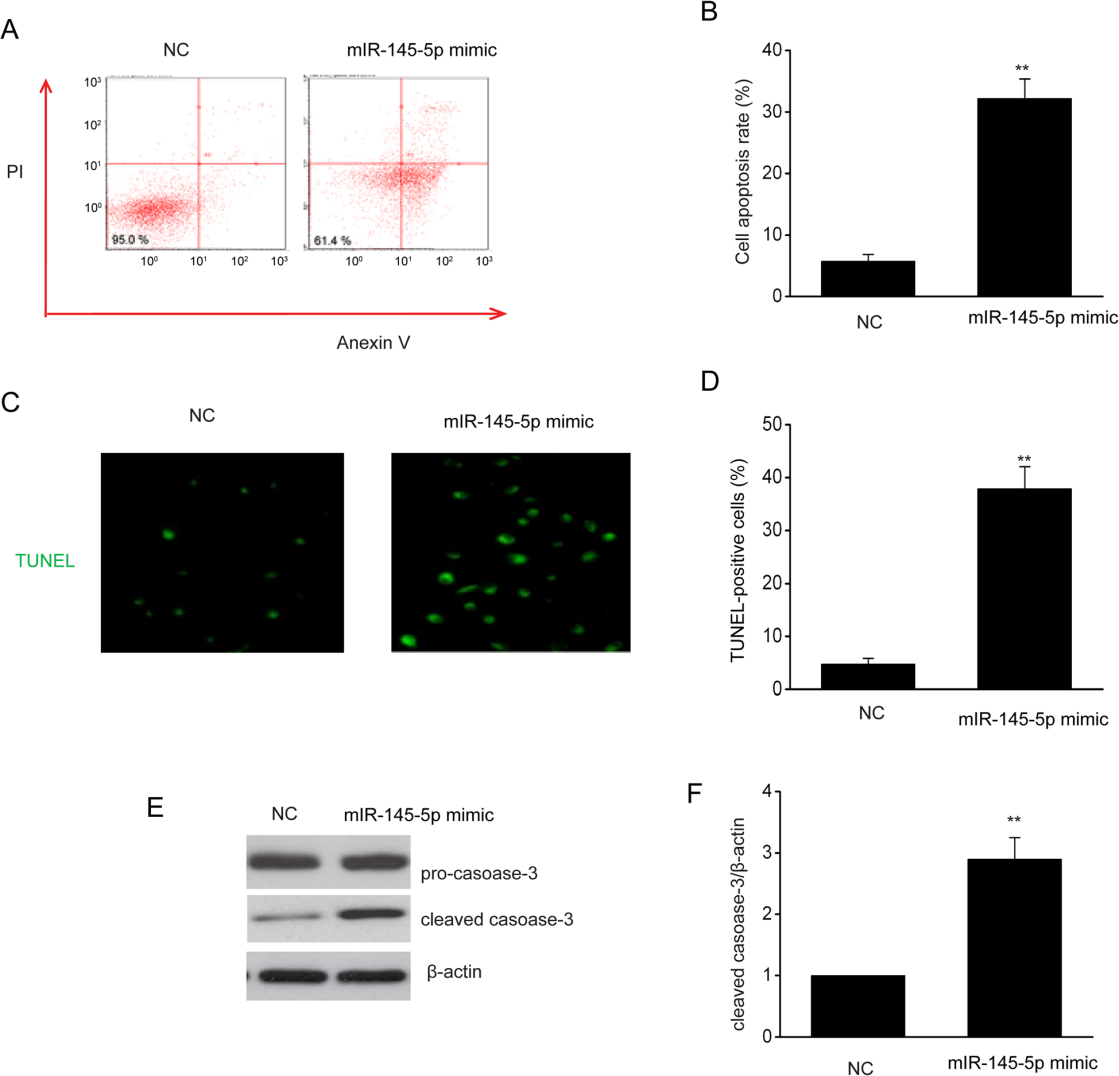
MiR-145-5p reinforced EOC cell death. **a-b**, MiR-145-5p reinforced cell death, as demonstrated USING FC. **c-d**, MiR-145-5p reinforced cell death, as confirmed using TUNEL test. **e-f**, Immunoblots (**e**) and the quantitative evaluation of caspase-3 (**f**) in SKOV-3 cells that were transfected for 24 h using miR-145-5p mimic or NC. Data are presented as means ± SEM, n = 3. ^**^P < 0.01, compared with the NC group

We investigated the contribution of miR-145-5p to EOC cell death through FC using Annexin V/PI staining. Cells that were transfected using miR-145-5p mimics demonstrated a noticeably elevated death rate in comparison with those in NC (Fig. 5a-b). Thereafter, cell death was verified using TUNEL test. Compared with those in NC, cells with positive TUNEL were remarkably reinforced in SKOV-3 cells following an excessive miR-145-5p expression (Fig. 5c-d), proving that miR-145-5p reinforced EOC cell death. In addition, the expression of cleaved capsase-3 was notably upregulated in miR-145-5p mimics transfected SKOV-3 cells (Fig. 5e-f).

### MiR-223 prohibited EOC cell migration

**Fig. 6:**
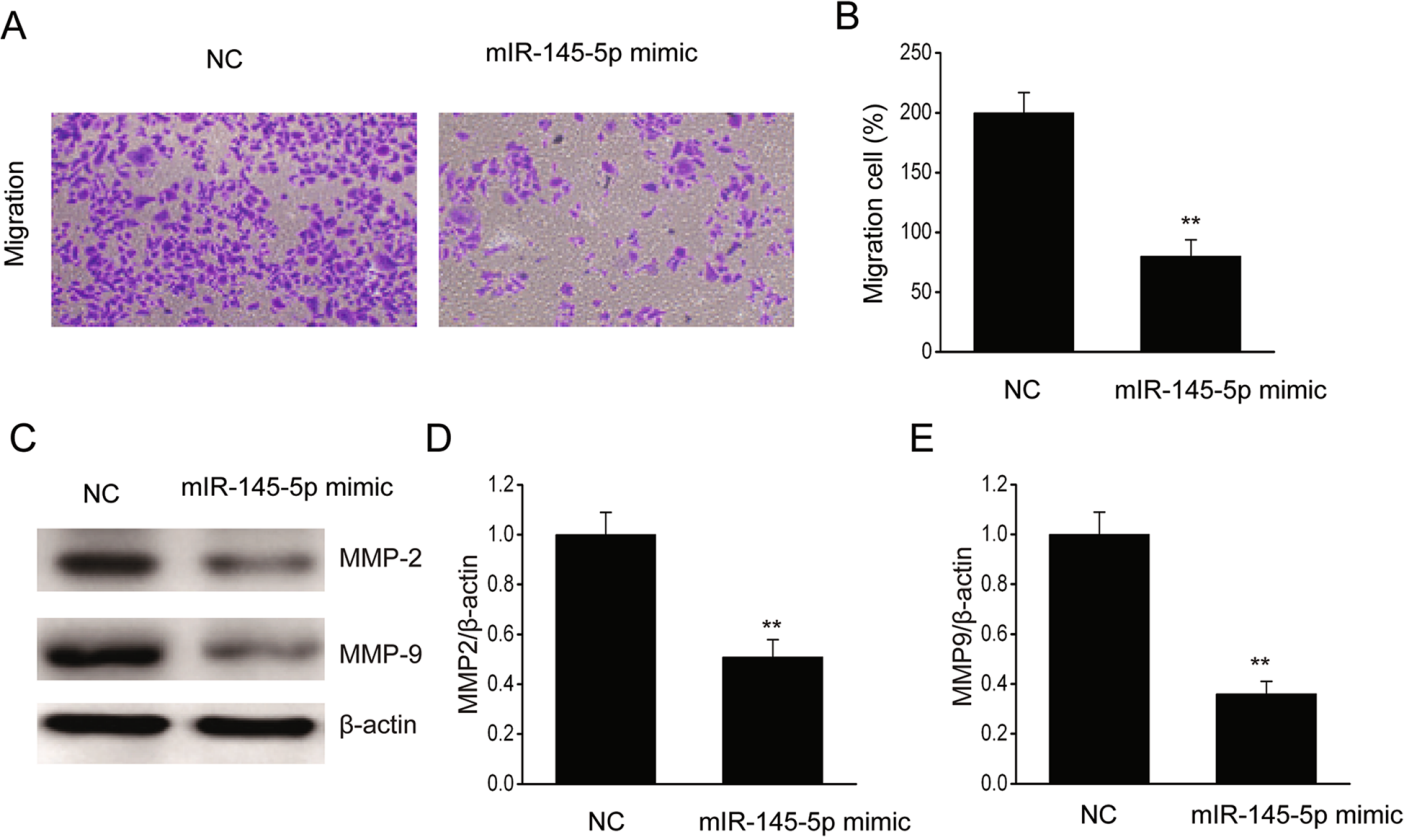
MiR-145-5p prohibited EOC cell migration. **a-b**, MiR-145-5p repressed migration, as evaluated via Transwell test. Representative images (**a**) and the quantitative evaluation of cell migration (**b**) are displayed. Scale bars = 100 μm. **c-e**, Immunoblots (**c**) and the quantitative evaluation of MMP-2 (**d**) and MMP-9 (**e**). Data are presented as means ± SEM, n = 3. ^**^P < 0.01, compared with the NC group

We determined the contribution of miR-145-5p to EOC cell migration using the Transwell test. Migration was noticeably suppressed via transfection using miR-145-5p mimics in SKOV-3 cells (Fig. 6a-b). Matrix metalloproteinases (MMPs) serve as the proteolytic enzymes of ECM relying on zinc, which is commonly used by cells via migration. MMP-2 and MMP-9 have closely been linked to the invasion of various malignancies [22, 23]. Hence, we investigated MMP-2 and MMP-9 translations to assess the contribution of miR-145-5p to EOC invasion. We found that excessive miR-145-5p expression remarkably repressed MMP-2 and MMP-9 translations (Fig. 6c-e), demonstrating that miR-145-5p reinforced EOC cell migration.

## Discussion

Our research proved that miR-145-5p was repressed in EOC tissues in comparison with that in the surrounding nonmalignant ones. Moreover, miR-145-5p reinforced EOC cell death and repressed EOC cell proliferation and migration via the direct modulation of SMAD4. Briefly, miR-145-5p could modulate EOC where SMAD4 had a contribution. This research presented an innovative understanding of the contribution of miR-145-5p to EOC etiology and indicated that miR-145-5p could serve as a promising target to treat EOC.

MiRNAs can repress or reinforce human malignancies [24, 25]. In particular, miR-145-5p displays an essential malignancy-repressing impact on malignancy progression [26, 27]. MiR-145-5p expression was repressed in several malignancies, such as non–small-cell lung cancer (NSCLC), ESCC, cervical cancer, and colorectal cancer, wherein it could repress malignancy generation [28–30]. Metadata proved that miR-145-5p is remarkably downregulated in NSCLC compared with that in normal tissues and could assist in a more accurate diagnosis in lung squamous cell carcinoma [31]. MiR-145 can repress various malignancies. Regarding gastric cancer, miR-145 modulates CD44 by directly targeting its 3′UTR [32]. Excessive miR-145 expression represses the migration, invasion, and proliferation of breast malignant cells via the downregulation of transforming growth factor-b expression [33]. In bladder malignancies, miR-145 expression is repressed. Its mimics repress the Warburg effect influenced directly by silencing Kruppel-like factor 4 (KLF4) [34]. Moreover, miR-145 inhibits resistance to oxaliplatin in colorectal cancer via GPR98 downregulation [35]. In ESCC, miR-145 expression is frequently repressed, whereas in esophageal EC9706 and ECA109 cells, its upregulation can prohibit proliferation and trigger cell death [36]. Wang et al. proved that excessive miR-145 expression remarkably represses ECA109 proliferation with the aid of pLVX-IZ-miR-145 vector and enhances the quantity of G2/M cells as well as cell death proportion [37]. In our research, the downregulation of miR-145-5p expression in EOC was verified. The activities and mechanism of the transcription modulation of miR-145-5p were subsequently investigated. Using MTT test, we found that miR-145-5p remarkably repressed EOC cell proliferation and migration. Furthermore, upon assessing the contribution of miR-145-5p to EOC cell death, we observed that miR-145-5p could reinforce EOC cell death. In summary, miR-145-5p could provide an essential target to treat EOC.

MiRNAs act by modulating the expression of target genes [38], and miR-145-5p represses malignancies via targeting some genes, such as mitogen-activated protein kinase 1, specificity protein 1, and nuclear factor-kB [28, 39, 40]. Furthermore, excessive miR-145-5p expression is modulated using circular or long noncoding RNAs [41, 42]. SMAD4 serves as a versatile essential modulator that functions with other transcription factors during TGF-β modulation in malignancy generation [43]. SMAD4 contributes to various cell reactions, and its malfunction is intimately linked to various malignancies, such as thyroid, pancreatic, prostate, and colorectal malignancies [44–46]. Therefore, in our research, miR-145-5p acted on SMAD4 3′UTR transcripts and downregulated SMAD4, thereby participating in the prohibition of EOC cell proliferation and migration as well as reinforcing EOC cell death.

## Conclusions

In conclusion, miR-145-5p repressed EOC generation and revealed an miR-145-5p/SMAD4 axis that repressed EOC cell proliferation and migration and reinforced EOC cell death. Therefore, excessive miR-145-5p expression could provide a promising strategy to treat EOC.

## Data Availability

The data used to support the findings of this study are available from the corresponding author upon request.
